# Multi-Omics Analysis Reveals the Adaptive Responses of *Lycoris aurea* to Arid Stress

**DOI:** 10.3390/biology15020195

**Published:** 2026-01-21

**Authors:** Mingxin Zhu, Zhaowentao Song, Yingzan Xie, Guanghua Liu, Youwei Zuo

**Affiliations:** 1 Key Laboratory of Research and Utilization of Ethnomedicinal Plant Resources of Hunan Province, Huaihua University, Huaihua 418008, China; 2 Key Laboratory of Eco-Environment in the Three Gorges Reservoir Region (Ministry of Education), School of Life Sciences, Southwest University, Chongqing 400715, China; 3 School of Biological Sciences, University of Auckland, Auckland 1010, New Zealand; 4 Chongqing Urban Ecosystem Observation and Research Station, Chongqing Academy of Forestry, National Forestry and Grassland Administration, Chongqing 400036, China

**Keywords:** *Lycoris aurea*, gene regulation, metabolome, soil properties

## Abstract

Understanding how plants respond to water limitation is increasingly important under accelerating climate change. *Lycoris aurea*, a widely distributed ornamental and medicinal bulbous plant, frequently inhabits environments with fluctuating soil moisture, yet its molecular drought-response mechanisms remain largely unexplored. In this study, we investigated *L. aurea* growing under field-based, in situ soil moisture regimes, comparing low (~20% soil water content) and high (~40% soil water content) conditions. We combined soil property assessments with high-resolution transcriptomic and untargeted metabolomic profiling to characterize the adaptive responses of bulb tissues under contrasting soil water conditions. Although total nitrogen, phosphorus, and potassium levels were comparable across treatments, soil moisture, representing the primary contrasting field condition, and soil pH, a correlated environmental factor, were significantly associated with variation in gene expression and metabolite accumulation (*p* < 0.05, *n* = 3). Transcriptome analyses identified a total of 1034 differentially expressed genes enriched in pathways related to amino acid metabolism, cuticle formation, cell wall modification, and osmotic adjustment. Metabolomic analysis identified a total of 1867 differentially expressed metabolites belonging to carboxylic acids and prenol lipids, showing alterations involved in amino acids, lipids, phenolic acids, and alkaloids associated with osmoprotection, membrane stabilization, and structural reinforcement under low soil moisture. Pathway-based integration analysis highlighted four core pathways, including “alanine, aspartate and glutamate metabolism” (*p* = 0.00371) and “cutin, suberine and wax biosynthesis” (*p* = 0.00873), as central hubs linking transcriptional regulation with metabolic reconfiguration. Gene-metabolite-soil correlation networks further demonstrated that drought adaptation arises from tightly coordinated biochemical and structural adjustments rather than shifts in nutrient acquisition. Together, this species-specific study provides a comprehensive multi-omics framework for understanding drought tolerance in *L. aurea*, reveals key molecular targets associated with plant resilience, and offers potential targets and insights for the conservation of drought-resilient *Lycoris* cultivars.

## Simple Summary

Drought is an increasing threat to plant survival, especially for non-model species growing under natural field conditions. In this study, we investigated how the bulbous plant *Lycoris aurea* responds to low soil moisture by integrating soil measurements with transcriptomic and metabolomic analyses. We found that changes in soil water availability, rather than nutrient levels, were closely associated with coordinated shifts in gene expression and metabolite profiles. These changes were mainly linked to amino acid metabolism, cell wall and cuticle reinforcement, and metabolic adjustments that support stress endurance. Our results suggest that *L. aurea* relies on coordinated molecular and metabolic strategies, centered on its bulb-based life history, to cope with water limitation. This work provides a field-based, multi-omics perspective on drought adaptation in a medicinal geophyte and offers a useful foundation for future physiological and functional studies.

## 1. Introduction

*Lycoris aurea* (Amaryllidaceae), commonly known as the golden spider lily, is a perennial bulbous plant widely cultivated for its striking golden-yellow inflorescences and its valuable Amaryllidaceae alkaloids, including lycorine and galanthamine, which have significant pharmacological properties [1,2]. Native to East and Southeast Asia-including China, Japan, Korea, and parts of Indochina, *L. aurea* typically inhabits forest margins, hillsides, riverbanks, and seasonally moist but periodically drought-prone environments [3]. In many parts of its habitat, *L. aurea* endures extended periods of limited precipitation, making drought an important selective force shaping its physiology and life-history traits. *L. aurea* is characterized by a robust bulb that serves as a long-term storage organ for water, carbohydrates, and nutrients [1,3]. This bulb enables the species to survive extended dry seasons and resume rapid growth once favorable conditions return. Such storage-based resilience is a hallmark of geophytic plants and suggests that *L. aurea* relies on tightly regulated molecular and metabolic strategies to cope with water limitation. Owing to its combination of ecological exposure to drought and specialized bulb-based physiology, *L. aurea* represents a suitable system for investigating the molecular mechanisms underlying drought adaptation in perennial bulbous plants.

Drought is one of the most pervasive environmental stresses limiting plant growth, productivity, and geographic distribution, particularly under the accelerating influence of global climate change [4,5]. Water deficit affects multiple physiological and biochemical processes, including photosynthesis, osmotic balance, and metabolic homeostasis, ultimately impairing plant development and survival [6,7]. Rather than uniformly affecting all plant types, water deficit imposes distinct constraints on perennial bulbous species, where survival depends less on continuous photosynthesis and more on the protection and metabolic regulation of underground storage organs [6,7]. In such plants, drought stress is closely associated with osmotic adjustment, carbon and nitrogen reallocation, and reinforcement of structural barriers that preserve bulb integrity during prolonged dry periods [8]. Previous studies in geophytes have shown that amino acid metabolism (especially proline and glutamate), lipid-derived cuticular components, and cell wall polysaccharide remodeling play central roles in drought tolerance, while growth-related metabolic pathways are often suppressed [9,10]. These responses help stabilize cellular membranes, reduce water loss, and maintain metabolic homeostasis within storage tissues, enabling regrowth when favorable conditions return [11,12]. However, whether and how these drought-adaptive mechanisms are coordinated at the transcriptomic and metabolomic levels in *L. aurea* remains largely unknown, limiting our understanding of stress resilience in this ecologically and medicinally important geophyte.

Some evidence suggests that *Lycoris* species exhibit moderate resilience to water deficit, potentially mediated by osmolyte accumulation, bulb-based water storage, and protective secondary metabolites [13,14]. However, these observations remain largely phenomenological and lack systems-level mechanistic resolution. In contrast, related bulbous genera within Amaryllidaceae and other geophytes such as *Narcissus*, *Allium*, or *Tulipa* have been shown to deploy coordinated transcriptional and metabolic programs involving amino acid cycling, cuticle reinforcement, and cell wall remodeling during drought [15,16,17]. Whether *L. aurea* shares these conserved strategies or exhibits distinct, species-specific regulatory architectures, particularly linked to its medicinal alkaloid metabolism and bulb physiology, remains unknown. Here, we address this gap by (i) characterizing drought-associated shifts in soil physicochemical properties relevant to *L. aurea* habitats, (ii) identifying drought-responsive genes and metabolites through integrated transcriptomic and metabolomic analyses, and (iii) resolving key co-regulated metabolic pathways that underpin drought adaptation. To test our hypotheses, we employed an integrative multi-omics framework combining soil physicochemical characterization with high-throughput transcriptomic and metabolomic profiling. This approach is essential for *L. aurea*, where drought adaptation is expected to emerge from cross-scale interactions among environment, gene regulation, and metabolite accumulation rather than from isolated molecular signals. Together, this study establishes an important multi-omics framework linking environmental elements with molecular and metabolic responses in *L. aurea*, providing a mechanistic basis for its drought resilience and advancing understanding of stress adaptation in plants.

## 2. Materials and Methods

### 2.1. Soil Sample Collection

To characterize the soil environment associated with *L. aurea*, we focused on a representative natural population located in Quannan County, Jiangxi Province (114°35′ E, 24°47′ N). This region maintains an annual mean temperature of approximately 18.6 °C, providing climatic conditions suitable for the species’ growth and survival. Soil moisture conditions were assessed in situ during the active growing season and reflect naturally occurring, stable field differences rather than experimentally imposed treatments. Field surveys consistently identified two contrasting soil water contents, approximately 20% (low moisture) and 40% (high moisture), which represent naturally maintained moisture regimes shaped by local topography and drainage rather than short-term fluctuations. Each soil moisture regime was represented by three independent biological replicates, with each replicate collected from a different, spatially separated mature plant (minimum distance > 10 m) to ensure statistical independence. For each replicate, soil was sampled at a radial distance of approximately 0.5 m from the bulb base and at a depth of 20–60 cm, corresponding to the primary rooting and bulb influence zone of *L. aurea*. This depth range was selected to encompass the active soil layer interacting with the bulb and perennial root system. Immediately after collection, soil samples were placed in insulated containers with ice packs and transported to the laboratory. In the laboratory, samples were manually cleaned by removing visible stones, coarse organic debris, and root fragments using sterile forceps, then gently homogenized and passed through a 2 mm stainless-steel sieve. Each sample was then divided into two portions: one stored at −80 °C for downstream integrative analyses with transcriptomic and metabolomic datasets, and the other used immediately for physicochemical measurements.

### 2.2. Soil Physical and Chemical Properties

Soil physicochemical properties were measured following well-established soil analysis protocols [18], with three independent biological replicates per group, and each measurement performed in technical duplicate. Soil water content (SWC) was determined gravimetrically by weighing fresh soil, oven-drying at 105 °C for 12 h, cooling in a desiccator, and reweighing to calculate moisture percentage. Soil pH was measured using a 1:2.5 soil-to-water suspension (*w*/*v*) after shaking for 30 min and allowing the mixture to settle. Nitrogen was quantified using the alkaline hydrolysis diffusion method, whereas phosphorus was extracted with 0.5 M NaHCO_3_ and measured colorimetrically at 882 nm. Potassium was extracted using 1.0 M NH_4_OAc (pH 7.0) and analyzed using flame photometry or atomic absorption spectrophotometry.

### 2.3. DNA Extraction and RNA Sequencing

Total RNA was extracted from bulb tissues collected from mature *L. aurea* plants at the same developmental stage, using the RNeasy Plant Mini Kit (Qiagen, Hilden, Germany) according to the manufacturer’s instructions. Bulb samples were harvested at the same time of day to minimize circadian variation, immediately frozen in liquid nitrogen, and stored at −80 °C until processing. For each biological replicate, 1.0–1.5 μg of total RNA was used as input for library construction. RNA concentration and purity (A260/A280 and A260/A230 ratios) were measured using a NanoDrop 2000 spectrophotometer (Thermo Fisher Scientific, Waltham, MA, USA), and RNA integrity was verified by 1% agarose gel electrophoresis and Agilent 2100 Bioanalyzer analysis (Agilent Technologies, Santa Clara, CA, USA), requiring RNA integrity number (RIN) values ≥ 7.0. Messenger RNA was enriched from total RNA using poly(A)+ selection with oligo(dT) magnetic beads, ensuring removal of rRNA and enrichment of protein-coding transcripts prior to library construction. Double-stranded cDNA was then end-repaired, 5′ phosphorylated, and 3′ adenylated to facilitate adaptor ligation. Sequencing libraries were constructed using the NEBNext^®^ Ultra™ RNA Library Prep Kit for Illumina^®^ (New England Biolabs, Ipswich, MA, USA; Cat. No. E7530), including ligation of NEBNext adaptors with unique index codes. Enriched libraries were amplified by limited-cycle PCR (12–15 cycles), purified, and assessed for quality using a Qubit 3.0 fluorometer (Invitrogen, Waltham, MA, USA) and Bioanalyzer 2100 (Agilent Technologies, Palo Alto, CA, USA) to confirm correct size distribution and concentration. Qualified libraries were pooled equimolarly and clustered using the TruSeq PE Cluster Kit v3-cBot-HS (Illumina, San Diego, CA, USA). Paired-end sequencing (150 bp × 2) was performed on an Illumina NovaSeq 6000 platform. Transcript abundance was quantified as fragments per kilobase of transcript per million mapped reads (FPKM) using StringTie v2.2.0 (http://ccb.jhu.edu/software/stringtie/, accessed on 19 February 2025).

### 2.4. RNA-Seq Dataset Processing and Analysis

Raw sequencing reads were subjected to stringent quality control prior to downstream analysis. Adapter sequences, poly-N reads, and low-quality bases were removed using Trimmomatic (v0.39). Clean reads were evaluated using FastQC (v0.11.9; https://www.bioinformatics.babraham.ac.uk/projects/fastqc/, accessed on 19 February 2025) and summarized using MultiQC (v1.14; https://multiqc.info/, accessed on 19 February 2025) to assess base quality, GC content, and sequence length distribution, ensuring the absence of systematic biases. Because no reference genome is currently available for *L. aurea*, high-quality reads from all samples were used for de novo transcriptome assembly using Trinity (v2.14.0; https://github.com/trinityrnaseq/trinityrnaseq, accessed on 19 February 2025), applying default parameters including a minimum k-mer coverage of 2. Assembly metrics (e.g., N50, mean contig length, and total assembled bases) were calculated using the built-in Trinity utility scripts. For functional annotation, predicted coding sequences were aligned to the NCBI non-redundant protein (Nr) database (https://www.ncbi.nlm.nih.gov, accessed on 19 February 2025) using BLASTx (BLAST+ v2.13.0) with an E-value cutoff of 1 × 10^−5^, and matches were also mapped to GO (Gene Ontology Consortium; http://geneontology.org/, accessed on 19 February 2025), KEGG (Kyoto Encyclopedia of Genes and Genomes; https://www.kegg.jp/, accessed on 19 February 2025), and COG/eggNOG (http://eggnogdb.embl.de/, accessed on 19 February 2025). For expression quantification, read alignments to the assembled transcriptome were performed using Bowtie2 (v2.5.1; http://bowtie-bio.sourceforge.net/bowtie2/, accessed on 19 February 2025), and gene- and isoform-level abundances were estimated with RSEM (v1.3.3; https://github.com/deweylab/RSEM, accessed on 19 February 2025), generating raw read counts, TPM, and FPKM values. TPM values were used for expression visualization and cross-sample comparison, whereas raw count data were used for differential expression analysis to avoid biases associated with FPKM normalization. Normalization and identification of differentially expressed genes (DEGs) between soil moisture treatments were conducted in R (v4.2.x; https://www.r-project.org/, accessed on 19 February 2025) using DESeq2 (v1.38.0), applying Benjamini–Hochberg correction to control the false discovery rate and retaining genes with adjusted *p*-value (FDR) ≤ 0.05 and |log2FoldChange| ≥ 2. GO and KEGG enrichment analyses of DEGs were performed with clusterProfiler (v4.6.0), using *q*-value ≤ 0.05 as the significance threshold, and enriched terms/pathways were visualized with the ggplot2 and pheatmap packages. For protein–protein interaction analysis, DEG-encoded proteins were queried against the STRING database (v11.5; https://string-db.org/, accessed on 19 February 2025) with a minimum interaction score of 0.7 (high confidence). Resulting networks were imported into Cytoscape (v3.10.0; https://cytoscape.org/, accessed on 19 February 2025), and densely connected subnetworks were extracted using the MCODE plugin with standard parameters (degree cutoff = 2, node score cutoff = 0.2, k-core = 2).

### 2.5. Metabolomic Sequencing and Data Analysis

Metabolite extraction was performed using approximately 80–100 mg of frozen *L. aurea* bulb tissue from each biological replicate. Samples were homogenized in 500 μL of 80% methanol containing internal standards, vortexed for 3 min, sonicated in ice water for 10 min, and precipitated at −20 °C for 30 min. The mixtures were centrifuged at 15,000× *g* for 20 min at 4 °C, and the resulting supernatants were filtered (0.22 μm) into LC-MS vials. A pooled QC sample was injected periodically throughout the run to monitor instrument stability. Chromatographic separation was carried out on a Thermo Ultimate 3000 UHPLC system, and metabolites were detected using a Thermo Q Exactive Orbitrap mass spectrometer operating in both positive and negative ESI modes. Full MS data were acquired at 70,000 resolutions with data-dependent MS/MS scans at 17,500 resolutions. Raw files were processed using Compound Discoverer v3.3 or XCMS, including peak detection, alignment, and normalization. Metabolite annotation was performed by matching accurate mass and MS/MS spectra against KEGG (https://www.kegg.jp/, accessed on 14 October 2024), HMDB, MassBank, and LIPID MAPS databases. Annotation confidence followed the Metabolomics Standards Initiative (MSI) guidelines, and most metabolites were assigned as putative annotations (Level 2) based on spectral similarity. Compounds uncommon or controversial in plant systems (e.g., deoxycholic acid (putative)) were therefore interpreted cautiously as putatively annotated features with structural similarity to known metabolites. Data were log_2_-transformed and analyzed using the ropls package in R for PCA and OPLS-DA modeling. OPLS-DA model quality was evaluated using cumulative R^2^X, R^2^Y, and Q^2^ values, and model robustness was further assessed by permutation testing using “ropls” package in R. Differentially accumulated metabolites (DEMs) were identified based on VIP ≥ 1, |log_2_FC| ≥ 1, and *p* ≤ 0.05. KEGG pathway enrichment was conducted using MetaboAnalyst 5.0, with significance defined at *q* ≤ 0.05. For integrative analysis, Spearman correlations (|r| ≥ 0.8, *p* ≤ 0.05) were calculated between transcriptomic DEGs and metabolic features, and shared KEGG pathways were visualized using KEGG Mapper and Cytoscape v3.10.0.

### 2.6. Statistical Analysis

All soil property, transcriptomic, and metabolomic data were analyzed using R software (v4.2.0) and SPSS 26.0. Soil variables and physiological indicators were tested for normality and homogeneity of variance prior to analysis, and differences between the two soil moisture treatments (20% vs. 40% SWC) were evaluated using Student’s *t*-tests at a significance threshold of *p* ≤ 0.05. For correlation-based integrative analyses, Spearman correlation coefficients were calculated using “Hmisc” package (v5.2, rcorr function in R), and multiple-comparison correction was applied using the Benjamini–Hochberg FDR method. Only correlations meeting both |r| ≥ 0.8 and FDR-adjusted *p* ≤ 0.05 were retained for downstream network construction and visualization. All graphs and heatmaps were generated using ggplot2, pheatmap, and MetaboAnalyst 5.0, and network visualizations were produced in Cytoscape v3.10.0.

## 3. Results

### 3.1. Soil Physicochemical Properties Under Contrasting Moisture Conditions

**Figure 1:**
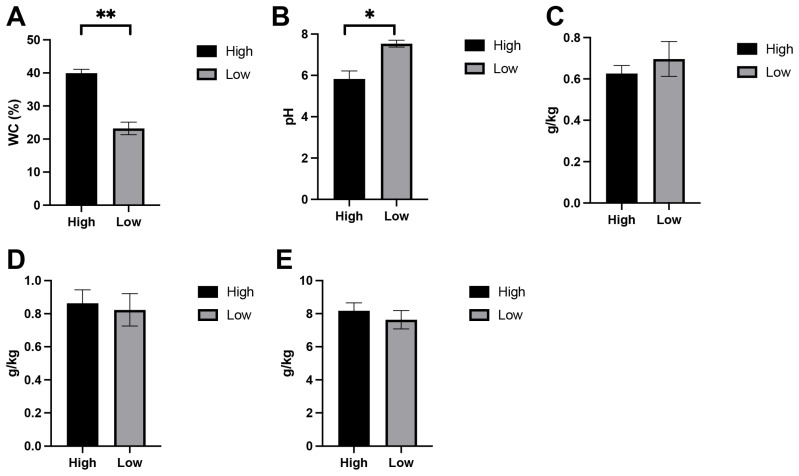
Soil physicochemical properties measured under high- and low-moisture conditions. Panels show (**A**) soil water content (WC, %), (**B**) soil pH, (**C**) total nitrogen (TN, g/kg), (**D**) available phosphorus (AP, g/kg), and (**E**) available potassium (AK, g/kg). Data represent mean ± SE for three biological replicates per treatment. */** (*p* < 0.05 or 0.01) indicate that there is a difference between the comparison.

Soils collected from the two moisture regimes exhibited clear differences in several key physicochemical parameters (Figure 1). As expected, the high-moisture treatment showed significantly greater soil water content than the low-moisture group (*p* < 0.01), confirming the effectiveness of the experimental gradient. Soil pH also differed between treatments, with slightly higher pH values observed in the wetter soils (*p* < 0.05). Major nutrient concentrations, including nitrogen, phosphorus, and potassium, showed no significant variation between treatments (*p* > 0.05).

### 3.2. Transcriptomic Responses of L. aurea to Arid Stress

**Figure 2:**
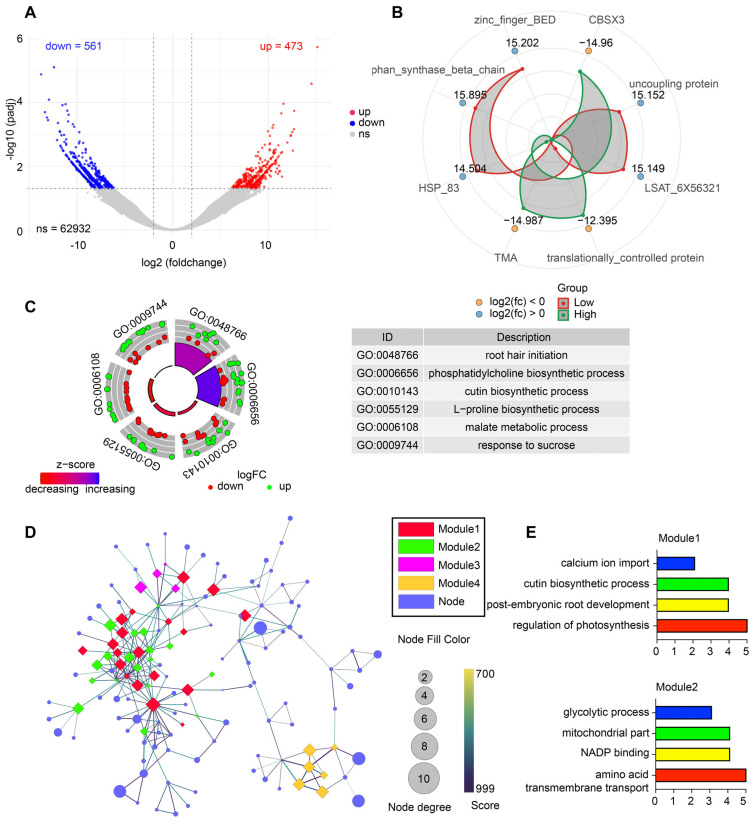
Transcriptomic responses of *L. aurea* under contrasting soil moisture conditions. (**A**) Volcano plot showing the distribution of differentially expressed genes (DEGs), including upregulated, downregulated, and non-significant genes. (**B**) Radar plot illustrating representative DEGs with log_2_ fold-change values in high- and low-moisture groups. (**C**) Circular GO enrichment visualization displaying significantly enriched Gene Ontology terms and associated up- and downregulated genes. The accompanying table summarizes selected enriched GO categories. (**D**) Protein–protein interaction (PPI) network of DEGs, highlighting four major MCODE-identified modules. Node color corresponds to module identity, and node size indicates degree. (**E**) Functional enrichment of the two major PPI modules, showing key biological processes associated with each module.

A total of 1034 DEGs were identified, including 473 upregulated and 561 downregulated transcripts under low soil moisture, while the majority of transcripts showed no significant change (Figure 2A). Radar plot visualization of representative DEGs highlighted strong moisture-responsive shifts in genes associated with stress protection, energy metabolism, and transcriptional regulation, including *CBSX3*, *HSP83*, and *TMA* (Figure 2B). GO enrichment of DEGs demonstrated that drought stress activated biological processes involved in root hair initiation, cutin biosynthetic process, L-proline biosynthesis, malate metabolism, and response to sucrose, many of which are associated with osmotic adjustment, cuticular reinforcement, and carbon remobilization (Figure 2C). Network analysis further resolved DEGs into four major co-expression modules (Figure 2D). Module 1 was enriched for drought-adaptive processes such as calcium ion import, regulation of photosynthesis, and post-embryonic root development. Module 2 contained genes linked to glycolytic process, mitochondrial function, NADP binding, and amino acid transport, indicating broad metabolic restructuring (Figure 2E).

### 3.3. Untargeted Metabolomic Profiling Reveals Distinct Metabolic Shifts Under Arid Stress

**Figure 3:**
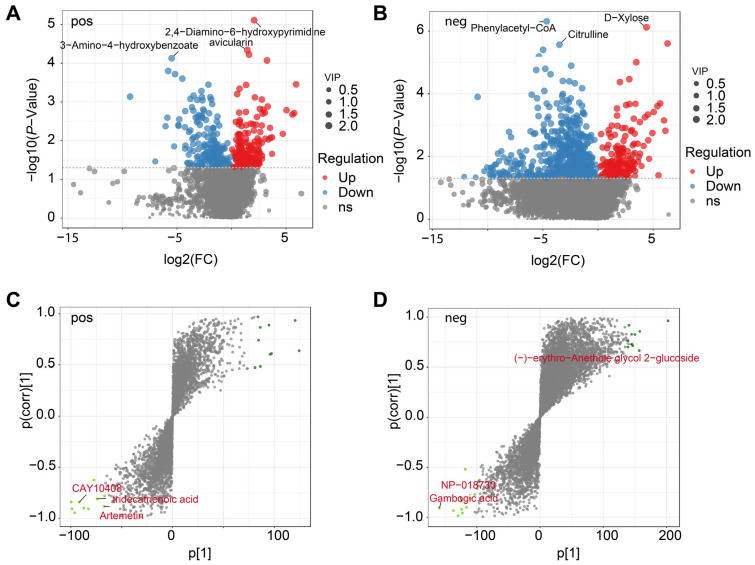
Metabolomic profiling of *L. aurea* under high- and low-moisture conditions. (**A**) Volcano plot of differentially expressed metabolites (DEMs) in the positive ion mode, showing upregulated, downregulated, and non-significant metabolites with variable importance in projection (VIP) scores indicated by point size. (**B**) Volcano plot of DEMs in the negative ion mode. (**C**) S-plot from OPLS-DA analysis in the positive ion mode highlighting metabolites contributing strongly to group separation. (**D**) S-plot from OPLS-DA analysis in the negative ion mode. Green points denote key metabolites with high variable weights in each model.

The study identified a total of 1867 DEMs in the comparison across positive and negative modes. In positive mode (Figure 3A), a total of 671 metabolites (377 up-regulated and 294 down-regulated) exhibited significant differential accumulation, with a subset strongly altered under low soil moisture (e.g., avicularin). Negative mode analysis identified a total of 1196 DEMs with 478 up-regulated and 718 down-regulated (Figure 3B), including prominent upregulated ions (e.g., D-xylose, quercitrin) and highly downregulated metabolites characterized by large negative fold changes (e.g., phenylacetyl-CoA, citrulline). To further evaluate treatment discrimination, OPLS-DA loading plots were generated (Figure 3C,D). Several ions contributed disproportionately to group separation, appearing as extreme loadings along both positive and negative axes. In positive mode (Figure 3C), key discriminant metabolites included CAY10408, tridecatrienoic acid, and artemetin, whereas negative mode analysis (Figure 3D) highlighted features such as (−)-erythro-Anethole glycol 2-glucoside, NP-018730, and gambogic acid.

**Figure 4:**
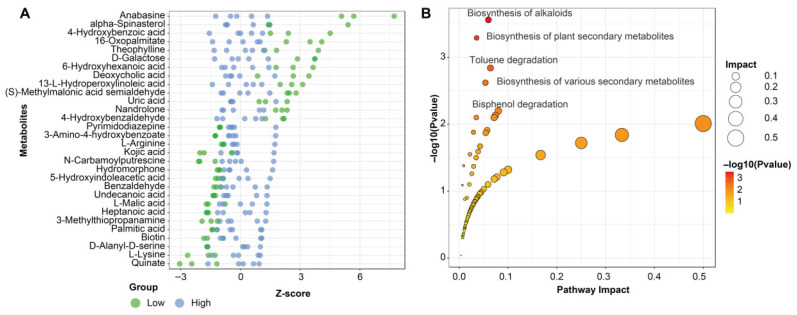
Differential metabolite profiles and pathway enrichment in *L. aurea* under contrasting soil moisture conditions. (**A**) Z-score plot showing the relative abundance patterns of selected differentially expressed metabolites across high- and low-moisture groups. (**B**) Metabolic pathway enrichment bubble plot based on KEGG annotations, displaying significantly enriched pathways, pathway impact values, and corresponding −log10 (*p*-value) for each pathway.

Several metabolites exhibited strong and consistent shifts between low- and high-moisture conditions (Figure 4A). Metabolites such as anabasine, alpha-spinasterol, 4-hydroxybenzoic acid, 16-oxopalmitate, theophylline, and D-galactose accumulated to higher levels under low soil moisture. Conversely, compounds including L-lysine, D-alanyl-D-serine, biotin, palmitic acid, 3-methylthiopropanamine, L-malate, and quinate were markedly reduced in the drought-exposed group. KEGG pathway enrichment further highlighted the metabolic processes most strongly affected by drought (Figure 4B). The top enriched pathways included biosynthesis of alkaloids, biosynthesis of plant secondary metabolites, and biosynthesis of various secondary metabolites, all exhibiting high pathway impact scores and low *p*-values. Additional pathways such as toluene degradation and bisphenol degradation were also enriched.

### 3.4. Integrated Transcriptomic and Metabolomic Analyses Identify Core Pathways Mediating Drought Adaptation

**Figure 5:**
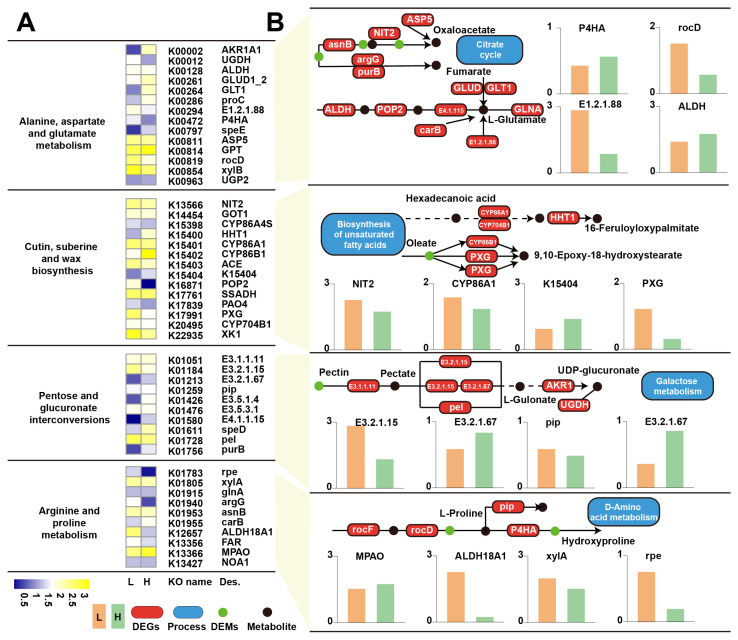
Integrated transcriptomic and metabolomic analysis of four drought-responsive KEGG pathways in *L. aurea*. (**A**) Heatmaps showing differential expression patterns of genes, enriched pathway processes, KEGG orthologs (KO), and associated metabolites across alanine/aspartate/glutamate metabolism (*p* = 0.00371), cutin/suberine/wax biosynthesis (*p* = 0.00873), pentose and glucuronate interconversions (*p* = 0.00091), and arginine/proline metabolism (*p* = 0.01392). Color gradients indicate relative expression or abundance levels under low- and high-moisture conditions. (**B**) Schematic pathway diagrams for each KEGG category, illustrating key genes, enzymes, and metabolites mapped from the integrated datasets, together with corresponding bar plots showing expression differences between moisture groups.

Combined KEGG mapping of DEGs and DEMs identified four pathways-alanine, aspartate and glutamate metabolism, cutin, suberine and wax biosynthesis, pentose and glucuronate interconversions, and arginine and proline metabolism-as the most strongly co-regulated modules in response to low soil moisture (Figure 5 and Table 1). In the amino acid and organic acid pathway, coordinated regulation of *GLUD*, *GLNA*, *GLT1*, *ASP5*, *NIT2*, and *POP2*, together with shifts in fumarate, oxaloacetate, L-glutamate, and L-aspartate, indicates rebalancing of carbon-nitrogen flux and TCA-linked amino acid cycling under drought. Genes involved in protective lipid polymer formation-including *CYP86A1*, *CYP704B1*, *CYP86B1*, *PXG*, and *P4HA*-were altered alongside increases in long-chain fatty acid derivatives such as hexadecanoic acid and 16-oxo-palmitate. The pentose and glucuronate interconversions pathway showed strong regulation of *UGDH*, *UGP2*, *xylA*, *pel*, and *AKR1*, matched with altered levels of UDP-glucuronate, L-gulonate, and related sugars. In the arginine and proline metabolism pathway, differential expression of *ALDH18A1*, *NOA1*, *MPAO*, *rpe*, and *P4HA*, together with accumulation of L-proline, hydroxyproline, and their precursors.

### 3.5. Gene-Metabolite-Soil Correlation Network Identifies Coordinated Drought-Response Modules

**Figure 6:**
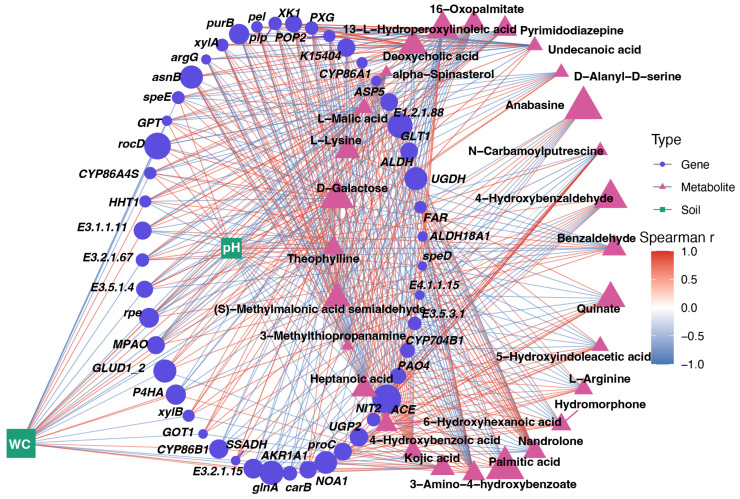
Integrated correlation network linking soil properties, drought-responsive genes, and metabolites in *L. aurea*. Nodes represent soil variables (green squares), genes (blue circles), and metabolites (pink triangles), with node size proportional to degree. Edges indicate significant Spearman’s correlations (|r| ≥ threshold), colored by correlation strength and direction (red = positive; blue = negative). The network visualizes multilevel interactions among soil water content, pH, transcriptomic responses, and metabolite profiles.

Correlation network analysis integrating soil variables, DEGs, and DEMs revealed a tightly connected regulatory structure linking soil moisture, pH, metabolic shifts, and drought-responsive gene expression in *L. aurea* (Figure 6). Soil WC and pH showed strong positive and negative correlations, respectively, with distinct sets of genes-including *UGDH*, *ALDH*, *GLT1*, *ASP5*, *GLUD1*/*2*, *NIT2*, *PXG*, *P4HA*, *rpe*, *xylA*/*xylB*, *argG*, *asnB*, *carB*, and *NOA1*-indicating that soil moisture acts as major environmental elements of transcriptional remodeling. These genes, many of which correspond to the four core pathways identified earlier (amino acid metabolism, cell wall modification, cutin-suberin-wax biosynthesis, and proline/arginine cycling), were strongly associated with drought-responsive metabolites such as 16-oxopalmitate, alpha-spinasterol, anabasine, 4-hydroxybenzoic acid, and N-carbamoylputrescine, which appeared on the highly correlated positive side of the network. Conversely, metabolites including L-malic acid, L-lysine, quinate, 3-methylthiopropanamine, and D-galactose were positioned within negatively correlated clusters.

## 4. Discussion

This multi-omics study provides a comprehensive characterization of drought-response strategies in *L. aurea*, integrating soil physicochemical properties, transcriptomic reprogramming, metabolomic adjustments, and combined pathway-level correlations. Despite stable nutrient conditions, reduced soil moisture acted as dominant environmental elements associated with gene expression, metabolite accumulation, and pathway activity. Through the identification of four major co-regulated pathways, our results highlight a coordinated molecular and biochemical framework that underpins drought adaptation in this species.

Notably, nitrogen, phosphorus, and potassium remained unchanged across soil moisture regimes, suggesting that drought tolerance in *L. aurea* is not achieved through altered nutrient acquisition but through metabolic plasticity within existing resource pools. This pattern aligns with observations in other perennial herbs and bulbous geophytes such as *Narcissus tazetta* and *Hemerocallis fulva*, where drought-triggered physiological alteration proceeded independently of nutrient supply [19,20], highlighting an inherent capacity for water-deficit acclimation driven by internal metabolic adjustments. Several studies in *Allium cepa* and *Aloe vera* also report that nutrient pools often remain stable during early- to mid-stage drought, while metabolic shifts-particularly in amino acids, soluble sugars, and lipids-are the predominant markers of stress response [21,22]. In *L. aurea*, this strategy is particularly adaptive given its perennial bulb habit, where survival depends on conserving internal reserves during prolonged dry periods rather than sustaining active uptake. Thus, water availability functions less as a limiting resource and more as a regulatory signal that reshapes metabolic priorities.

Several metabolites strongly accumulated under drought, such as anabasine, alpha-spinasterol, 4-hydroxybenzoic acid, and long-chain fatty acid derivatives. The enrichment of these compound classes is consistent with metabolic shifts commonly associated with stress endurance, including adjustments related to cellular protection and surface barrier maintenance [23,24]. Similar drought-induced metabolic signatures have been reported in *Lycoris radiata* and *Panax notoginseng*, where elevated phenolic acids, sterols, oxylipins, and alkaloids stabilize cellular structures and reduce water loss [14,25]. Conversely, the depletion of malate, lysine, quinate, and soluble sugars indicates a deliberate suppression of growth-oriented carbon fluxes in favor of defense and maintenance, a trade-off that is particularly relevant for geophytes whose fitness depends on preserving underground organs across seasons [26,27,28]. Notably, the strong enrichment of cutin, suberine, and wax biosynthesis genes indicating potential enhanced extracellular barrier formation as documented in drought-resilient geophytes [29]. Hence, these coordinated metabolic and transcriptional changes collectively reflect a drought-adaptation strategy centered on bulb protection and long-term survival rather than rapid growth recovery.

The coordinated enrichment of alanine, aspartate and glutamate metabolism, pentose and glucuronate interconversions, arginine and proline metabolism, and cutin, suberin and wax biosynthesis reflects an integrated drought-response strategy. Amino acid metabolism appears to function as a central metabolic hub, linking nitrogen remobilization with carbon flux redistribution to sustain basal energy production and redox balance under water limitation [30,31]. This metabolic buffering likely supports continued TCA cycle activity while constraining growth-related carbon expenditure [32]. Similarly, the activation of pentose and glucuronate interconversions-driven by genes such as *UGDH*, *UGP2*, and pel-indicates active remodeling of cell wall polysaccharides. Such cell wall reprogramming is increasingly recognized as a central stress-adaptive strategy, as modifications in pectin demethylesterification, hemicellulose turnover, and wall stiffness directly regulate cellular water retention, mechanical stability, and stress signaling [33,34,35]. The upregulation of cutin, suberin, and wax biosynthesis complements these internal metabolic adjustments by strengthening extracellular diffusion barriers, thereby reducing transpirational and evaporative water loss [36,37]. Together, these pathways form a coordinated trade-off system in which metabolic flexibility, structural reinforcement, and surface protection operate synergistically to preserve bulb integrity during drought.

Although this study establishes a foundational multi-omics framework for understanding drought adaptation in *L. aurea*, several avenues warrant further investigation. First, because our analyses were conducted on a single natural population under two in situ soil moisture regimes, caution is warranted when extrapolating these findings across the species’ full geographic range or to other environmental contexts. The absence of controlled drought treatments further limits the ability to disentangle drought-specific effects from site-dependent environmental variation, and our conclusions should therefore be interpreted as associative rather than causative. Nevertheless, this field-based design captures ecologically realistic water-limitation scenarios and provides a valuable baseline for future comparative work. In addition, no direct plant physiological measurements (e.g., leaf water status, stomatal conductance, or hormone levels) were obtained, and drought-related responses are inferred primarily from soil conditions and omics-level associations. Direct anatomical and histochemical analyses of root, shoot, and leaf tissues would be valuable to validate the cell wall remodeling and protective barrier reinforcement inferred from transcriptomic and metabolomic data. Detailed examination of cell wall composition, lignification, pectin modification, and tissue-level structural changes before and after drought exposure would strengthen the mechanistic link between molecular regulation and plant morphology. The relatively small sample size (*n* = 3 per condition) also constrains statistical power, and expanding replication across populations and environments would improve robustness and generalizability. Temporal dynamics of drought responses remain unexplored; profiling time-course changes in gene expression and metabolites would clarify primary versus downstream regulatory events. Functional experiments, such as gene silencing or overexpression of key regulators, would enable causal links between pathways and drought tolerance to be validated. Comparative analyses involving different *Lycoris* species or natural populations from varying climates could reveal evolutionary divergence in drought-resilience mechanisms. Finally, integration of hormonal signaling, root-microbiome interactions, and proteomic profiling would provide a more comprehensive systems-level understanding of how *L. aurea* copes with increasingly arid habitats, supporting future breeding or conservation efforts.

## 5. Conclusions

This multi-omics investigation provides a comprehensive molecular and biochemical framework describing how *L. aurea* adapts to arid soil conditions. Despite largely stable nutrient availability, soil moisture emerged as an environmental factor linked to transcriptomic and metabolomic reprogramming. Integrated analyses revealed four core pathways that potentially underpin drought resilience through enhanced osmotic regulation, strengthened protective barriers, and targeted remodeling of nitrogen and carbon metabolism. The coordinated shifts in genes and metabolites highlight an adaptive strategy centered on metabolic flexibility and structural fortification. While the relationships identified here are correlative, they offer a coherent framework for understanding drought-associated molecular responses in *L. aurea* and provide a foundation for future functional studies aimed at validating candidate pathways and traits relevant to stress resilience.

## Figures and Tables

**Table 1:** Differentially expressed genes associated with four key drought-responsive KEGG pathways.

Ko ID	KEGG Description	Name	log2FC	*q*-Value
ko00250	Alanine, aspartate and glutamate metabolism	*ALDH*	1.34	0.01241
		*E1.2.1.88*	−2.13	0.00583
		*P4HA*	1.23	0.00139
		*rocD*	−1.87	0.00912
ko00073	Cutin, suberine, and wax biosynthesis	*NIT2*	−2.13	0.00084
		*CYP86A1*	−1.98	0.00139
		*K15404*	1.76	0.03922
		*PXG*	−3.14	0.00018
ko00040	Pentose and glucuronate interconversions	*E3.2.1.67*	1.65	0.00148
		*pip*	−1.76	0.00204
		*E4.1.1.15*	−2.13	0.00463
		*purB*	2.31	0.00618
ko00330	Arginine and proline metabolism	*rpe*	−2.11	0.00081
		*xylA*	−1.77	0.00104
		*ALDH18A1*	−3.41	0.00003
		*MPAO*	1.44	0.04191

## Data Availability

The sequencing datasets have been deposited in the NCBI Sequence Read Archive (SRA) under the BioProject accession number PRJNA1100760 (RNA-seq). The metabolomics data have been submitted to MetaboLights under accession MTBLS9960. All additional data supporting the findings of this study are available from the corresponding author upon reasonable request.
